# Evidence-based family planning services among publicly funded providers in Texas

**DOI:** 10.1186/s12913-022-08889-0

**Published:** 2022-12-09

**Authors:** Shetal Vohra-Gupta, Elizabeth Ela, Elsa Vizcarra, Liana J. Petruzzi, Kristine Hopkins, Joseph E. Potter, Kari White

**Affiliations:** 1 Steve Hicks School of Social Work, The University of Texas at Austin, 1925 San Jacinto Blvd, Austin, TX 78712 USA; 2 Population Research Center, University of Texas at Austin, 305 E. 23Rd Street, Austin, TX 78712 USA; 3 Texas Policy Evaluation Project, The University of Texas at Austin, 116 Inner Campus Dr., Austin, TX 78712 USA

**Keywords:** Health policy, Women’s health, Reproductive health services, Family planning, Contraception, Health surveys

## Abstract

**Background:**

Healthy Texas Women (HTW) is a fee-for-service family planning program that excludes affiliates of abortion providers. The HTW network includes providers who participate in Title X or the state Family Planning Program (FPP) and primary care providers without additional family planning funding (HTW-only). The objective of this study is to compare client volume and use of evidence-based practices among HTW providers.

**Methods:**

Client volume was determined from administrative data on unduplicated HTW clients served in fiscal year (FY) 2017. A sample of 114 HTW providers, stratified by region, completed a 2018 survey about contraceptive methods offered, adherence to evidence-based contraceptive provision, barriers to offering IUDs and implants, and counseling/referrals for pregnant patients. Differences by funding source were assessed using t-tests and chi-square tests.

**Results:**

Although HTW-only providers served 58% of HTW clients, most (72%) saw < 50 clients in FY2017. Only 5% of HTW providers received Title X or FPP funding, but 46% served ≥ 500 HTW clients. HTW-only providers were less likely than Title X providers to offer hormonal IUDs (70% vs. 92%) and implants (66% vs 96%); offer same-day placement of IUDs (21% vs 79%) and implants (21% vs 83%); and allow patients to delay cervical cancer screening when initiating contraception (58% vs 83%; all *p* < 0.05). There were few provider-level differences in counseling/referrals for unplanned pregnancy (*p* > 0.05).

**Conclusions:**

HTW-only providers served fewer clients and were less likely to follow evidence-based practices. Program modifications that strengthen the provider network and quality of care are needed to support family planning services for low-income Texans.

## Introduction

Since 2007, Texas hasconsecutively implemented three different fee-for-service family planning programs that have provided low-income, uninsured U.S. resident women between 18 and 45 years of age with contraception, screening for cervical cancer and sexually transmitted infections, and related reproductive health services. The re-organization of the fee-for-service program over a short time period was prompted by the state’s loss of federal Medicaid matching funds in 2013 when Texas excluded qualified providers affiliated with abortion care (e.g., Planned Parenthood) from participating, as well as the consolidation of other state-funded family planning programs [1]. Texas officials claimed that other participating providers, as well as many newly recruited to the provider network, would be able to serve clients who had to change their source of care [2].

In the initial years following these programmatic revisions, both client enrollment and the number of clients using services declined by 24% and 39% respectively, even as the provider network grew [2, 3]. Additionally, providers with less reproductive healthcare experience did not offer enrolled clients the same range of services as specialized family planning organizations, and there was a marked decrease in the provision of intrauterine devices (IUDs), contraceptive implants and injectable contraceptives [1, 4, 5]. A 2015 qualitative assessment further found that new contractors in one of the second-generation programs lacked capacity to offer robust family planning services, including limited training to place IUDs and contraceptive implants and familiarity with evidence-based protocols for providing contraception [6]. There was also variability in providers’ practices surrounding counseling and referrals for unplanned pregnancy in that not all providers offered comprehensive counseling and referrals for unplanned pregnancy. This finding was due in part to a requirement that participants in the state-funded fee-for-service program had to annually attest that they did not “promote abortion” and were not affiliated with an organization that provided abortion [7]. Limited research has assessed whether provider capacity has strengthened in the ensuing years and whether barriers to offering evidence-based care persisted in the provider network – particularly among individual or group practices that account for a large share of enrolled providers.

Despite little evidence that care improved under fully state-funded family planning programs, in January 2020, the federal Department of Health and Human Services approved Texas’ application for federal matching funds for the Healthy Texas Women’s (HTW) program [8], the third fee-for-service program established in 2016 that still excludes Planned Parenthood. This allows Texas to receive an estimated $70 million annually in federal funding, which accounts for 90% of the program’s costs. In November 2020, Texas also excluded Planned Parenthood from its full benefit Medicaid program and has continued to receive federal funding. The unprecedented decision to provide federal funding to programs that do not allow patients freedom of choice of provider may offer an opening for other states that have previously been blocked from excluding Planned Parenthood from their Medicaid programs [9]. These policy shifts point to the need to assess HTW provider performance and quality of care to ensure patients’ timely access to evidence-based services.

For this study, we analyzed state administrative data on the number of clients HTW providers served and data from a survey of HTW providers about their clinical practices related to the provision of reversible contraceptive methods and referrals for unplanned pregnancy. We considered differences between HTW providers that only participated in the fee-for-service program (HTW-only providers) and those that also received other sources of family planning funding, such as federal Title X funds or the state-funded Family Planning Program (FPP), which provides family planning services to low-income women (and men) < 65 years of age, regardless of immigration status. Providers who participate in one or both of these programs may have greater experience and financial support for providing contraceptive services, and therefore may be more likely to offer evidence-based care [6]. Identifying differences among providers could point to programmatic changes that may be needed to reduce patient barriers to care.

## Methods

### Administrative data on clients served

We obtained aggregated administrative data, collected by the Texas Health and Human Services Commission, on the number of unduplicated HTW clients served by all participating providers during fiscal year (FY) 2017 (September 2016 – August 2017).Clients who received any service covered by the program, including but not limited to a contraceptive method, were counted. Since providers could work at multiple locations, and unique locations may have more than one provider, we grouped providers into practice sites and organizational systems based on addresses and organizational names. We removed providers who were not located in Texas or who were unlikely to provide contraceptive services, such as dental clinics, imaging centers, and laboratories. Using information on providers’ other sources of family planning funding, we categorized providers as HTW and Title-X-funded providers, HTW and FPP-funded providers, and HTW-only providers. We included 13 HTW providers that received both Title X and FPP funds in the HTW and Title X category, given differences in Title X’s programmatic guidelines with respect to requiring confidential services, counseling and referrals for unplanned pregnancy and administrative support.

### Provider survey

The Texas Health and Human Services Commission provided us with a list of providers enrolled in the HTW program as of November 2017. Following the procedures described above, we grouped providers into practice sites and removed those that were not located in Texas or were unlikely to provide contraceptive services.

From 1,053 sites based in Texas, we selected all practices and organizations that received Title X or FPP funding since 2013 (*n*= 65). These organizations represent diverse service delivery models (e.g., Federally Qualified Health Centers [FQHCs], health departments, specialized family planning providers) and typically serve a large number of family planning clients [5, 6]. We also sampled 150 HTW-only providers, who were primarily clinicians at individual or group practices. For this sample, we stratified providers across Texas’ eight health service regions and determined the number of sampled providers in each region based on probability proportional to size, where size was the number of reproductive-aged women from the 2015 American Community Survey [10], the most recent five-year estimates available at the time of the study. Then, within each region, we used a random number generator to draw the HTW-only provider sample.

Given prior reports of inaccuracies in the provider list [11], we verified sampled providers’ contact information and participation in HTW via online searches and phone calls to practice locations. Providers that could not be reached after multiple attempts or no longer participated in HTW were excluded and replaced with another provider located in the same health service region.

In May 2018, we mailed sampled providers a letter inviting them to complete an online survey and sent an email if an email address was available. All letters included a two-dollar incentive, and providers who completed the survey were entered into a raffle to receive one of ten $50 Amazon gift cards. We called practice locations and sent follow-up letters and emails encouraging providers to participate. A total of 114 HTW providers answered the survey.

The survey collected information on the type of practice (e.g., private practice, FQHC), provider specialty (e.g., women’s health, family medicine), family planning client volume and the types of contraceptive methods they provided onsite. Because HTW aimed to expand the provider base to include private practice clinicians whose main focus may not be family planning, we also assessed providers’ use of evidence-based practices. Specifically, we asked how likely they were to allow patients to initiate a hormonal method at any time in their menstrual cycle if they are reasonably sure they are not pregnant (i.e., quick start) and whether they allowed patients to delay physical exams before obtaining a method [12–14]. We also asked whether their practice offered same-day initiation of IUDs and implants; barriers to offering IUDs and implants (e.g., clinician training, stocking devices, and reimbursement); and provider beliefs about the suitability of these methods for teens and women who have not had a child, regardless of whether they provided these methods onsite. We based these items on questions used in other surveys of provider practices in California and Texas [15–17].

Finally, we asked respondents about their practices related to pregnancy options counseling and referral for patients experiencing an unplanned pregnancy. We hypothesized that some providers may be reluctant to offer comprehensive counseling and referrals, despite professional recommendations, because HTW providers must attest to the fact they that do not perform abortions and are not affiliated with an abortion provider to participate in the program [7, 18, 19]. Specifically, respondents reported on a four-point Likert scale how likely they were to discuss abortion, adoption, and continuing pregnancy and, separately, how likely they were to refer patients to providers that offer abortion, adoption services, or prenatal care if a patient requested additional information. The study was approved by the authors’ university institutional review board.

### Analysis

From the administrative data, we computed the median (interquartile range [IQR]) number of unduplicated clients served in FY2017. Following an examination of the distribution of the data, we also categorized the client totals as < 50 clients, 50–249, 250–499 and ≥ 500. We compared differences in clients served according to sources of family planning funding (i.e., HTW only, HTW and Title X, and HTW and FPP), using Kruskal Wallis and Fischer exact tests.

Using the survey data, we computed the distribution of provider and practice characteristics and calculated the percentage of providers offering oral contraceptives, injectables, IUDs, implants, and emergency contraception (EC). We counted the number of contraceptive methods that providers offered onsite. We also computed the percentage of providers who reported barriers to offering IUDs and implants onsite, were very likely or somewhat likely to use quick start protocols, offered same-day placement of IUDs and implants, and considered teens (15–19 years old) and nulliparous (women who have not had children) patients to be appropriate candidates for long-acting methods. Finally, we examined the percentage of providers who offered comprehensive pregnancy options counseling and direct referrals for abortion, adoption, and prenatal care. Following other studies [20], we combined the responses “very likely” and “somewhat likely” to describe providers’ reports of their likelihood to discuss abortion, adoption, and parenting with patients experiencing unplanned pregnancy. We also combined responses to indicate whether provider were very or somewhat likely to provide direct referrals or a list of agencies for abortion, adoption, and prenatal care. We assessed differences according to provider funding source using t-tests for continuous variables and chi-squared tests for categorical variables. All analyses were conducted using Stata 15.

## Results

### Administrative data on clients served

**Fig. 1:**
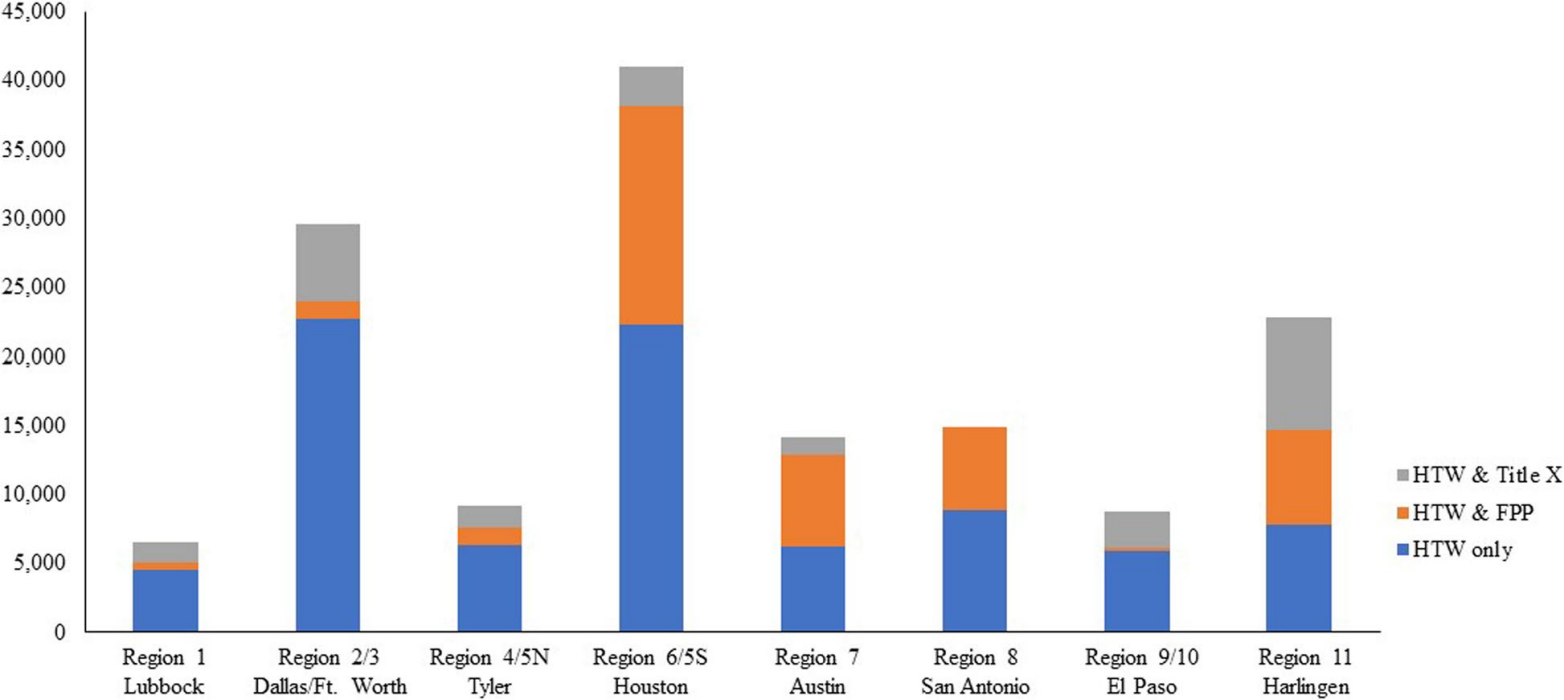
Distribution of unduplicated clients served in the Healthy Texas Women program (FY17), by provides’ family planning funding source and healthy service region

After excluding providers who were not located in Texas or who were unlikely to provide contraceptive services, we identified 1,266 HTW practice sites in the administrative data. Of these, 24 received Title X funding, 39 received FPP funding and 1,203 participated in the HTW fee-for-service program only (Table 1). Together, these providers served a total of 147,002 family planning clients in FY2017. HTW-only providers served 57.6% of all clients but overall client volume was lower among these providers, compared to those who received other sources of family planning funding. For example, only 2% (*n* = 28) of HTW-only providers served ≥ 500 clients in FY2017, compared to nearly half of the HTW providers who received Title X or FPP funding (*p* < 0.001). The majority (72%) of HTW-only providers served < 50 clients. There was also regional variation in provider participation in the different family planning funding sources and clients served, with higher numbers of unduplicated clients served in the health service regions that were home to the state’s largest metropolitan areas (i.e., Dallas/Fort Worth and Houston; Fig. 1).

**Table 1:** Number of unduplicated clients served in HTW program (FY 2017), by providers’ family planning funding source

	**Providers’ sources of family planning funding**		
**Clients served**	**HTW only (** ***n*** ** = 1,203)**	**HTW & FPP (** ***n*** ** = 39)**	**HTW & Title X**^a^ **(*n* = 24)**
Total clients, n	84,682	38,703	23,617
Median (IQR)*	14 (3, 58)	346 (174, 1,246)	409 (228, 1,176)
Distribution of clients, n providers (%)^§^			
< 50	870 (72.3)	8 (20.5)	0
50–249	260 (21.6)	7 (18.0)	7 (29.1)
250–499	45 (3.7)	6 (15.4)	6 (25.0)
≥ 500	28 (2.3)	18 (46.2)	11 (45.8)

Data source: Healthy Texas Women program data on clients served in fiscal year 2017 (September 2016-August 2017)

*FPP* Family Planning Program, *IQR* Interquartile range

^a^Includes 13 organizations receiving funding from HTW, FPP and Title X

^*^Kruskal Wallis test *p*-value < .001

^§^Fischer’s exact test *p*-value < .001

### Provider survey

Of the 215 practice locations invited to participate in the survey, 114 (53%) completed it. Most survey respondents were physicians or advanced practice clinicians (Table 2). Nearly all respondents had worked at their practice for at least one year, and over 60% had worked at their practice for at least 5 years. Among these practices, the most common specialty was women’s health, family planning, or gynecology (64%), followed by family medicine, internal medicine, or general practice (31%). In addition to participating in HTW, 21% of these providers also received federal Title X funding and another 22% received FPP funds.

**Table 2:** Characteristics of HTW Provider Survey Respondents

*Respondent Characteristics*	%
Gender	
Female	69.0
Male	31.0
Training	
Physician	47.0
Advanced Practice Clinician	41.0
Nurse/Other	12.0
Position at organization	
Clinician	52.0
Clinical/Medical Director	30.0
Other	18.0
Time working at organization	
Less than 1 year	7.0
1 to 4 years	31.0
5 to 9 years	20.0
10 or more years	42.0
*Practice Characteristics*	
Specialty	
Women’s health, family planning, or obstetrics and gynecology	64.0
Family medicine, internal medicine, or general practice	31.0
Other	5.0
Family planning funding sources	
HTW only	57.0
HTW & Family Planning Program (FPP)	22.0
HTW & Title X^a^	21.0

Data source: Healthy Texas Women provider survey, 2018 (*n* = 114)

^a^Includes 14 organizations receiving funding from HTW, Family Planning Program and Title X

On average, HTW providers offered 4.7 reversible contraceptive methods onsite. Nearly all providers offered contraceptive injections onsite, and the majority also offered hormonal and copper IUDs and implants (Table 3). HTW-only providers were less likely than those with Title X funding to offer hormonal IUDs, copper IUDs, and implants onsite (all *p* < 0.05).

**Table 3:** Contraceptive methods offered, by Healthy Texas Women providers’ funding source

	All providers (*n* = 114)	Provider funding source			
		HTW Only (*n* = 65)	HTW & FPP (*n* = 25)	HTW & Title X (*n* = 24)	*p*-value†
*Methods Offered Onsite, %*					
Hormonal IUD	79.0	70.0	88.0	92.0	0.041
Copper IUD	73.0	65.0	76.0	91.0	0.044
Implant	78.0	66.0	92.0	96.0	0.002
Injection	97.0	94.0	100.0	100.0	0.210
Oral Contraceptive Pills	44.0	17.0	68.0	92.0	< 0.001
Patch	22.0	9.0	44.0	33.0	0.001
Vaginal Ring	38.0	13.0	61.0	83.0	< 0.001
*Emergency Contraception, %*					
Likely/very likely to counsel emergency contraception if patient had unprotected sex in past 5 days	76.0	65.0	84.0	100.0	0.001
Practice offers emergency contraception	75.0	66.0	76.0	100.0	0.004

Data source: Healthy Texas Women provider survey, 2018 (*n* = 114)

^†^*p*-values obtained from chi-square tests

Common barriers to offering hormonal IUDs, copper IUDs, and implants included: denied claims for reimbursement (21%-25% across methods), inadequate reimbursement (20%-24%), and slow reimbursement (13%-15%). Additionally, 21% of respondents reported lack of clinician training as a barrier to offering copper and hormonal IUDs. Although most barriers did not differ by provider type, HTW-only providers were more likely than other provider types to indicate that having insufficient devices onsite was a barrier to offering long-acting reversible contraception (LARC) methods. Nearly half (47%) of HTW-only providers reported having insufficient hormonal IUDs available onsite, versus 4% of FPP providers and 24% of Title X providers (*p* < 0.01). Results were similar for the copper IUD and implant (not shown).

Combined hormonal methods (oral contraceptive pill, patch, and vaginal ring) were available onsite at fewer practices than IUDs and implants. HTW-only providers were the least likely to offer oral contraceptive pills onsite (17%), followed by FPP providers (68%) and Title X providers (92%) (*p* < 0.001). Approximately three quarters of providers said they were likely or very likely to counsel patients who reported having unprotected sex within the past five days about EC and indicated that their practice offered EC. Two-thirds of HTW-only providers responding to the survey reported counseling about and offering EC, whereas all providers with Title X funding did so (*p* < 0.01).

HTW-only providers were less likely to report using evidence-based practices that facilitate contraceptive access than providers with other funding sources (Table 4). Overall, 58% of HTW-only providers allowed patients to delay or forgo cervical cancer screening, compared to 83% of providers with Title X funding. Additionally, 22% of HTW-only providers offered same-day placements of IUDs and implants, whereas 79% of providers with Title X funding offered same-day IUD placement and 83% offered same day implant placement. Approximately 20% of all providers, regardless of funding sources, did not consider teens 15 to 19 years old or nulliparous women to be suitable candidates for copper and hormonal IUDs (not shown).

**Table 4:** Evidence-based contraceptive provision practices, by Healthy Texas Women providers’ funding source

Evidence-based practice, %	All providers (*n* = 113)	Provider funding source			
		HTW Only (*n* = 65)	HTW & FPP (*n* = 24)	HTW & Title X (*n* = 24)	*p*-value†
Can delay/forgo cervical cancer screening to start a method	68.0	59.0	80.0	83.0	0.030
Provider very/somewhat likely to recommend quick start	82.0	72.0	88.0	100.0	0.007
Same-day IUD placement available	39.0	22.0	46.0	79.0	< 0.001
Same-day implant placement available	43.0	22.0	60.0	83.0	< 0.001

Data source: Healthy Texas Women provider survey, 2018 (*n* = 113)

^†^*p*-values obtained from chi-square tests

Nearly half (48%) of providers indicated that they would be very or somewhat likely to provide comprehensive options counseling (i.e., counseling about continuing the pregnancy, adoption, and abortion) to patients experiencing an unplanned pregnancy (Table 5). Overall, 42% of HTW-only providers and 62% of HTW providers at Title X- funded organizations reported comprehensive counseling (*p* = 0.09). Nearly all providers would counsel patients about continuing the pregnancy, and most (82%) were likely to discuss adoption as an option, but only half reported they were likely to discuss abortion if the patient requested additional information. Similarly, referrals for prenatal care were nearly universal, but only 58% reported they were likely to refer or provide a list of adoption agencies and even fewer (38%) were likely to refer or provide a list of facilities providing abortion (*p* > 0.05).

**Table 5:** Counseling and referrals for unplanned pregnancy, by Healthy Texas Women providers’ funding source

When counseling patient with unplanned pregnancy, provider is likely or very likely to…		Provider funding source			
	All providers (*n* = 112)	HTW Only (*n* = 64)	HTW & FPP (*n* = 24)	HTW & Title X (*n* = 24)	*p*-value†
Provide comprehensive options counseling (discuss all three options)	48.0	42.0	50.0	63.0	0.232
Discuss continuing pregnancy as an option	95.0	92.0	100.0	96.0	0.335
Discuss adoption as an option	82.0	84.0	79.0	79.0	0.776
Discuss abortion as an option	51.0	45.0	50.0	67.0	0.202
Provide direct referral for all three options	30.0	28.0	25.0	38.0	0.597
Refer for prenatal care or schedule a prenatal visit	96.0	95.0	100.0	96.0	0.564
Refer or provide a list of agencies that provide adoption	58.0	58.0	50.0	67.0	0.504
Refer or provide a list of agencies that provide abortion	38.0	38.0	33.0	42.0	0.837

Data source: Healthy Texas Women provider survey, 2018 (*n* = 112)

^†^*p*-values obtained from chi-square tests

## Discussion

Following changes to Texas’ publicly funded family planning programs, previous studies demonstrated that women with low incomes experienced disruptions in their access to care, and new providers often lacked capacity to effectively offer patients evidence-based family planning services [1, 4, 21]. The present analyses update these findings and indicate that, four years after Planned Parenthood was excluded from Texas’ fee-for-service family planning program in 2013, the network of providers, particularly those who only participated in HTW, continued to face constraints with respect to number of clients served, scope, and quality of services.

State administrative data demonstrated an increase in participating providers in the fee-for-service program after Planned Parenthood was excluded [3]. Yet, a closer examination revealed that the many HTW-only individual and group practices that made up a large part of the provider network in 2017 served fewer clients on average than providers that received additional sources of family planning funding. According to our analysis, HTW providers at organizations receiving Title X or FPP funding served more than 40% of all program clients in 2017 but accounted for only 5% of all HTW network providers. That a small number of providers continues to play an outsized role in serving program clients suggests that high-volume providers remain critical for sustaining the program and increasing the number of network providers may not be sufficient for expanding clients’ access to evidence-based care. Women may also have faced difficulties locating a source for care due to inaccuracies in the provider list [11], contributing to relatively low client volume at participating individual and group practices.

Additionally, we found considerable variation in the scope and quality of family planning services delivered according to providers’ receipt of other family planning programs. HTW-only providers were less likely than those with Title X funding to offer hormonal IUDs, copper IUDs and implants onsite. Our finding that HTW-only providers were less likely to offer IUDs and implants onsite, compared to organizations with Title X funding, is similar to results from a national survey showing onsite availability of these devices is more common among publicly funded family planning providers with Title X funding [22]. HTW-only providers were also less likely to offer same-day IUD and implant placements. These results suggest that without other funding sources, HTW-only providers may be unable to purchase devices in advance to offer same-day placements for patients who desire them. This could directly impact access to IUDs and implants, particularly for low-income women, as transportation barriers or limited paid time off could hinder patients’ ability to return to the clinic for IUD or implant placement. However, other factors that may limit patients’ access to the full range of methods were more similar across providers in our survey, regardless of funding sources. Specifically, one in five providers did not consider teens and nulliparous women to be appropriate candidates for IUDs. Previous studies found that more than one in four Texas women with low incomes are interested in using these methods [23, 24]; therefore, efforts are needed to increase provider awareness of patient eligibility and reduce unnecessary access barriers.

Finally, we found that less than half of providers offered comprehensive pregnancy options counseling and referrals, and referrals for abortion care were particularly limited. These findings are consistent with prior qualitative research from Texas [6] and national data [20, 25]. This may reflect providers’ assumption about patients’ pregnancy desires. However, in Texas specifically, lack of comprehensive options counseling and referrals may also be related to the fact that providers participating in state-funded family planning programs, including HTW, are required to sign an attestation form stating that they will not provide or promote abortion services, including offering any information about abortion to women experiencing unplanned pregnancies [7]. Providers may interpret these restrictions to include a prohibition against providing comprehensive counseling on pregnancy options. Such a requirement is inconsistent with professional medical guidelines about essential components of quality care that can help patients make an informed decision, obtain timely services and patients’ preferences to receive unbiased information about their options [18, 19].

Together these findings point to several opportunities that could strengthen the provider network and quality of care in the HTW program. This is especially relevant following the US Supreme Court’s decision in *Dobbs v. Jackson Women’s Health Organization*, which overturned *Roe v. Wade*, and implementation of a total abortion ban in Texas. Making these family planning programs and services more robust for people living on low incomes becomes essential, as they will now face greater obstacles in getting abortion care. Ensuring a robust provider network will involve efforts to sustain those organizations that see a large volume of family planning patients, as well as strengthening training and education for providers at small-volume sites; this could include reducing barriers to adequate reimbursement and offering funding supports to facilitate patient enrollment and use of program services. Also, program modifications that allow all HTW providers to purchase and stock IUDs and implants in advance of patients’ request would improve timely initiation of these methods for those who want them [26]. Relatedly, removing the annual certification requirement that HTW providers do not provide or promote abortion care could increase other family planning providers’ participation in the program, not only those affiliated with Planned Parenthood, and reduce information barriers for patients who do not want to continue their pregnancies.

Although we surveyed providers from across the state, the response rate was higher among large-volume providers that received other sources of family planning funding (Title X and FPP) than the response rate among those serving fewer clients, which tended to be HTW-only providers (75% vs 43%). Therefore, practice patterns among individual and group practice providers may be different than those reflected here. Additionally, characteristics of providers and program participation may have changed from the 2017 administrative data and provider list used. However, the number of billing providers in HTW has remained relatively stable since 2017 [3], suggesting there may have been few shifts in the provider network. Also, a recent report of mystery clients calls to Texas Medicaid providers indicates that few are able to provide timely, evidenced based contraceptive care, suggesting limitations in the network of providers persist for people living on low incomes [27]. Following the onset of the 2020 COVID-19 pandemic, there has been a decrease in patient volume and services across providers [28]. In addition, providers may be more reticent to counsel and refer patients to abortion care following the implementation of Texas Senate Bill 8 (SB8) based on misinterpretations of the law’s restrictions about ‘aiding and abetting’ a prohibited abortion [29]. Therefore, future research is needed to identify differential impacts of these changes across provider types.

Despite these limitations, this study provides new evidence that the family planning safety net may still be falling short of serving Texas women with low incomes years after the state excluded Planned Parenthood. Seemingly, the path to rebuilding the network may be slow when relying on smaller-volume providers who may have less familiarity with evidence-based reproductive health care. This information should be considered when making administrative decisions about providing federal support for family planning programs that exclude qualified providers.

## Data Availability

The datasets generated and/or analyzed during the current study are not publicly available due to the inability to fully anonymize the data and organizational responses given the small number of providers who receive Title X and FPP funding but are available from the corresponding author on reasonable request.
